# Wet bio-amniotic membrane plugging combined with transplantation in the treatment of small corneal perforations

**DOI:** 10.5935/0004-2749.2022-0328

**Published:** 2024-02-23

**Authors:** Yingxin Chen, Chenxi Lv, Minghong Gao, Zhiling Liu, Ruiyao Gao

**Affiliations:** 1 Department of Ophthalmology, The General Hospital of Northern Theater Command, Shenyang, China

**Keywords:** Amnion, Transplantation, Amniotic membrane, Keratoplasty, penetrating, Corneal perforation, Wet bio-amniotic membrane plugging, Wet bio-amniotic membrane transplantation

## Abstract

**Purpose:**

Wet bio-amniotic membrane plugging combined with transplantation is a novel option that combined amniotic membrane plugging with amniotic membrane transplantation for the treatment of small corneal perforations. This study aimed to evaluate the efficacy of wet bio-amniotic membrane plugging in the treatment of small corneal perforations and compared it with that of the penetrating keratoplasty procedure.

**Methods:**

Forty patients (41 eyes) with small corneal perforations <3 mm in diameter treated at our hospital between July 2018 and January 2021 were retrospectively included. Among them, 21 eyes were treated with wet bio-amniotic membrane plugging (wet bio-amniotic membrane plugging group), and 20 eyes were treated with penetrating keratoplasty procedure (penetrating keratoplasty procedure group). The best-corrected visual acuity, anterior chamber formation, corneal thickness, primary disease control, postoperative complications, and graft survival rate were assessed.

**Results:**

No significant difference in baseline characteristics was found between the wet bio-amniotic membrane plugging and penetrating keratoplasty procedure groups (p>0.05). The postoperative control rates of primary diseases in the wet bio-amniotic membrane plugging and penetrating keratoplasty procedure groups were 95.2% and 90.0%, respectively (p=0.481). Visual acuity was improved 6 months after the operation in the wet bio-amniotic membrane plugging group and was improved at postoperative 1 month in the penetrating keratoplasty procedure group. The formation time of the anterior chamber in the wet bio-amniotic membrane plugging group was significantly shorter than that in the penetrating keratoplasty procedure group (p=0.023). The corneal thickness of the two groups significantly increased 12 months after the operation; however, the degree of thickening in the penetrating keratoplasty procedure group was higher than that in the wet bio-amniotic membrane plugging group (p<0.001). During the follow-up, postoperative complications were not different between the two groups (p>0.999).

**Conclusion:**

The results suggest that wet bio-amniotic membrane plugging is effective and safe in the treatment of small corneal perforations. Thus, it can be used as an emergency treatment alternative to penetrating keratoplasty procedure for small corneal perforations.

## INTRODUCTION

According to the statistics of the World Health Organization, approximately 20 million of the 39 million people living with blindness had corneal disease^(1)^. In China, corneal diseases are the second leading cause of blindness after cataracts^(2)^. If patients with corneal disea-ses are not treated promptly, worsening corneal epithelial defects and corneal stroma dissolution may cause corneal ulcers or perforation. Severe cases may result in blindness or enucleation^(3)^. These findings indicate that timely treatment to seal the perforation is important to maintain eye integrity and prevent infection.

Corneal perforations must be treated immediately, and appropriate treatment options depend mainly on the size and location of the corneal perforations and status of the primary disease^(4)^. For small perforations, tissue adhesives and corneal bandage lens can be used to promote healing of the perforation^(5-7)^. However, these methods have several limitations. In general, patients with corneal perforations require surgical intervention. The penetrating keratoplasty procedure (PKP) is commonly used for corneal perforations^(4)^. Despite the satisfactory success rate of allograft transplantation, infective corneal perforations can easily cause graft rejection, eventually leading to graft failure^(8)^. Moreover, the demand for corneas is far greater than its supply in clinical practice^(9)^. Thus, ophthalmologists have tried to use amniotic membranes to treat corneal perforations.

The amniotic membrane is the innermost layer of fetal membranes, which have unique properties such as facilitating the healing of damaged epithelial cells and reducing the inflammatory response and ingrowth of new blood vessels^(10)^. The first application of amniotic membrane for therapeutic ophthalmology was done by De Rötth in 1940^(11)^. In the treatment of small corneal perforations, previous studies have demonstrated that amniotic membrane plugging and transplantation are good alternative approach^(12,13)^. This approach is based on plugging the perforation with amniotic membranes and then transplanting a large piece of the amniotic membrane to cover the entire cornea. However, most of the amniotic membranes used clinically in hospitals are self-made with no clear guidelines, which may cause safety problems. With the in-depth understanding of the biological characteristics of the amniotic membrane, a wet bio-amniotic membrane was launched in China in December 2019. The wet bio-amniotic membrane was made from fresh amniotic membranes by rapid sampling, cutting, and irradiation sterilization, which can effectively retain various natural active components of the amniotic membrane and has the natural effect of anti-inflammation and antiscaring^(14)^. Meanwhile, aseptic preparation and virus inactivation ensure the biosafety of wet bio-amniotic membranes, which has the advantages of safety and convenience in ocular surface repair surgery^(15)^. In China, the efficacy and safety of wet bio-amniotic membranes in the treatment of small corneal perforations (diameter <3 mm) have not been evaluated.

In this study, we aimed to evaluate the efficacy of wet bio-amniotic membrane plugging (AM-P) combined with transplantation in the treatment of small corneal perforations and then compare it with that of the PKP. Our results demonstrated that the AM-P combined with transplantation may be employed as an emergency treat-ment alternative to PKP for small corneal perforations.

## METHODS

### Patients

We retrospectively analyzed patients with small corneal perforations who underwent surgery at our hospital between July 2018 and January 2021. According to surgical methods, patients were divided into the AM-P group and the PKP group.

The inclusion criteria were as follows: (1) diagnosis of corneal perforations and failed conservative treatment; (2) corneal perforation diameter within 3 mm; (3) age 16-80 years; (4) cooperation with an eye examination; (5) follow-up at least 12 months; and (6) presence of fundus disease affecting postoperative visual acuity. The exclusion criteria were as follows: (1) successful conservative treatment and (2) presence of fundus diseases that affects postoperative vision, such as age-related macular degeneration, optic atrophy, and diabetic retinopathy.

### Material sources

The amniotic membranes used in the AM-P group were Ruixiufu Wet Bio-amniotic Membrane (Guangzhou Ruitai Biotechnology Co., Ltd, Guang, China). The donor corneas used in the PKP group were from healthy and fresh cadaveric eyes collected within 2 h after death. After treatment, corneal grafts that fully meet safety standards were used for PKP surgery. The quality and safety monitoring items included donor disease spectrum examination, hepatitis B virus, hepatitis C virus, syphilis, and acquired immunodeficiency syndrome.

### Surgical technique

**Figure 1:**
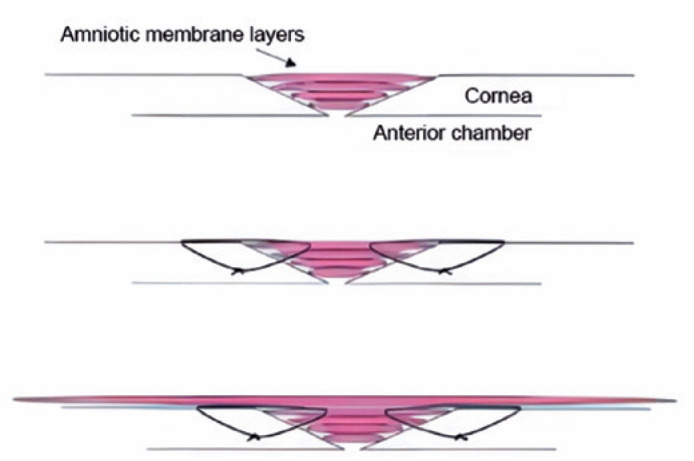
Surgical scheme for small corneal perforations. Note: A multilayer wet bio-amniotic membrane was inserted into the ulcer perforation as a filling material; thereafter, a larger amniotic membrane was used to cover the entire cornea.

AM-P surgery. Eyelash cutting, eyelid margin cleaning, conjunctival sac flushing, and lacrimal duct flushing were performed before the operation. After routine disinfection and topical anesthesia, corneal ulcer lesions and adjacent necrotic tissues were excised, and the limbus was punctured at 2 o’clock. The iris trapped in the corneal perforation area was restored by an iris restorer, and then a balanced salt solution was injected to form the anterior chamber. A wet bio-amniotic membrane was taken and folded into multilayers according to the ulcer depth, and wet bio-amniotic membrane plugging was performed. The basement membrane of the outermost amniotic membrane was upward, which was intermittently sutured with 10-0 sutures around the ulcer and perforation area. Then, amniotic membrane transplantation was performed. Another larger amniotic membrane was laid on the surface of the cornea, and the 10-0 sutures were sutured 1 mm inside the corneal limbus for a continuous circle. The surgical scheme for small corneal perforations is shown in figure 1. Until the amniotic membrane was attached to the cornea, the bandage contact lens was covered. After the operation, the eye was coated with ointment, covered with an aseptic dressing, and wrapped.

PKP surgery. At 30 min before surgery, pilocarpine nitrate eye drops (once for 5 min, a total of six times, Shandong Bausch & Lomb Freda Pharmaceutical Co., Ltd., Jinan, China) was used to induce miosis, and a methazolamide tablet (0.5 mg) (Suzhou Homesun Pharmaceutical Co., Ltd., Suzhou, China) was taken orally to reduce the intraocular pressure. After routine disinfection, proparacaine hydrochloride eye drops (Alcain, Alcon, Fort Worth, TX, USA) were given for topical anesthesia, followed by injection of 2% lidocaine (Tiansheng Pharmaceutical Group Co., Ltd., Shiyan, China) for retrobulbar block and softening of the eyeball for 10 min. According to the condition of the operating eye, a trephine drill with an appropriate diameter was perpendicular to the corneal surface and rotated clockwise to drill the implant bed. On the basis of the implant bed, a trephine drill larger than 0.25 mm in diameter of the implant bed was used to drill the donor cornea and obtain the implant. Then, 10-0 sutures was used for four stitches interrupted at 3, 6, 9, and 12 o’clock positions to fix the graft, and then a total of 16 interrupted stitches were made to bury the knot. To reconstruct the anterior chamber, the puncture port was made at the limbus at 2 points, and a balanced saline solution was injected. After surgery, the eye was coated with ointment and wrapped.

### Postoperative management

All patients received rational use of drugs according to each condition. Specifically, all patients were covered with a bandage contact lens after surgery, and the eye was coated with ointment, covered with an aseptic dressing, and wrapped. Then, all patients received a daily routine dressing change. If the anterior chamber was formed, the eye can be opened, and anti-infection, anti--inflammatory, and corneal epithelial growth promotion treatments were given. At postoperative week 1 to month 6, the amniotic membrane sutures were removed in the AM-P group. However, depending on the condition, the corneal sutures were removed 3-12 months after PKP. Corneal bandage lenses were replaced every 21 days until all the sutures were removed.

### Follow-up

All patients were followed up for at least 12 months. During the follow-up, the primary disease in the AM-P group was successfully controlled if the following criteria were met: complete corneal epithelium, negative Seidel test, and absence of recurrence. In the PKP group, corneal graft transparency indicated success in controlling the primary disease^(16)^. BCVA, expressed as a logarithm of minimal angle of resolution (logMAR), was employed to assess postoperative recovery. In addition, anterior chamber formation, corneal thickness, and complications were recorded.

### Statistical analysis

Data were analyzed using IBM SPSS Statistics version 26.0 (IBM Corp., Armonk, NY, USA). The enumeration data were expressed as number or percentage and analyzed by the chi-square test (n≥5) or Fisher’s exact test (n<5). Measurement data were expressed as mean ± standard deviation. Data conforming to a normal distribution were analyzed using Student’s t-test, whereas data with non-normal distribution were analyzed by the Wilcoxon rank-sum test. The significance of the BCVA and corneal thickness was compared by the two-way analysis of variance (ANOVA), followed by the Bonferroni test. Graft survival was estimated by Kaplan-Meier followed by the log-rank test. P<0.05 was considered statistically significant.

## RESULTS

### Baseline characteristics

A total of 40 patients (41 eyes) with small corneal perforations were enrolled in this study, including 29 men and 12 women, with an average age of 55.05 ± 14.83 years. Among them, 21 eyes were in the AM-P group and 20 eyes were in the PKP group. The baseline characteristics of the AM-P and PKP groups are shown in table 1. No obvious differences in age, sex, perforation diameter, BCVA, and primary diseases were noted between the groups (p>0.05).

**Table 1:** Baseline characteristics

Parameters	AM-P group (n=21)	PKP group (n=20)	p-value
Age, years	53.43 ± 18.13	56.75 ± 10.52	0.476
Sex (male/female)	16/5	13/7	0.431
Perforation diameter (mm)	1.80 ± 1.04	1.58 ± 0.55	0.400
BCVA (logMAR)	1.23 ± 0.86	1.71 ± 0.71	0.069
Primary diseases, n (%)			0.241
Viral keratitis	5 (23.8)	8 (40.0)	-
Bacterial keratitis	2 (9.50)	5 (25.0)	-
Fungal keratitis	3 (14.3)	3 (15.0)	-
Mooren ulcer	2 (9.50)	2 (10.0)	-
Corneal foreign body	8 (38.1)	2 (10.0)	-
Severe dry eye	1 (4.80)	0 (0.00)	-

### Visual acuity

As shown in table 2, the BCVA was not different between the two groups (F=0.024, p=0.877); however, an obvious time effect was observed (F=29.85, p≤0.001). Importantly, a significant interaction between time and group was also found (F=3.414, p=0.010). In the between-group comparison, the BCVA in the AM-P group was remarkably decreased 6 months after the operation and then tended to be stable. However, the visual acuity in the PKP group improved at postoperative month 1. These results indicated that the visual acuity in the PKP group improved more quickly than that in the AM-P group.

**Table 2:** Comparison of the BCVA and corneal thickness at different times

	AM-P group (n=21)	PKP group (n=20)
**BCVA at different times**		
Pre-operation	1.23 ± 0.86	1.71 ± 0.71
Postoperative month 1	1.31 ± 0.59	1.09 ± 0.22^*^
Postoperative month 3	0.98 ± 0.49	0.85 ± 0.22^*^
Postoperative month 6	0.61 ± 0.31^*^	0.60 ± 0.16^*^
Postoperative month 12	0.55 ± 0.37^*^	0.47 ± 0.14^*^
Corneal thickness		
Preoperation (µm)	138.7 ± 37.15	123.6 ± 16.79
Postoperative month 12 (µm)	465.1 ± 52.89^*^	528.0 ± 38.14^*^#

* p<0.05= within-group difference vs. pre-operation;

# p<0.05= between-group difference vs. the AM-P group. AM-P= wet bio-amniotic membrane plugging combined with transplantation; BCVA= best-corrected visual acuity; PKP= penetrating keratoplasty procedure.

### Anterior chamber formation

Anterior chambers were formed in all patients within 3 days after the operation. In the AM-P group, the anterior chamber in the 18 eyes formed on postoperative day 1. In the PKP group, five eyes formed anterior chambers on postoperative day 3, six eyes formed anterior chambers on postoperative day 2, and nine eyes formed anterior chambers on postoperative day 1. In addition, a statistical difference was found between the two groups (p=0.023) (Table 3).

**Table 3:** Comparison of anterior chamber formation, controlling primary diseases, and complications between the AM-P group and the PKP group

	AM-P group (n=21)	PKP group (n=20)	p-value
Anterior chamber formation			
Postoperative day 1 (n, %)	18 (85.7)	9 (45.0)	-
Postoperative day 2 (n, %)	2 (9.50)	6 (30.0)	-
Postoperative day 3 (n, %)	1 (4.80)	5 (25.0)	0.023
Analysis of controlling primary diseases			
Success (n, %)	20 (95.2)	18 (90.0)	-
Failure (n, %)	1 (4.80)	2 (10.0)	0.481
Postoperative complications			
Secondary infection (n, %)	1 (4.8)	0 (0.0)	-
High intraocular pressure (n, %)	1 (4.8)	0 (0.0)	-
Graft immune rejection (n, %)	0 (0.0)	1 (5.0)	>0.999

AM-P= wet bio-amniotic membrane plugging combined with transplantation; PKP= penetrating keratoplasty procedure.

### Corneal thickness

**Figure 2:**
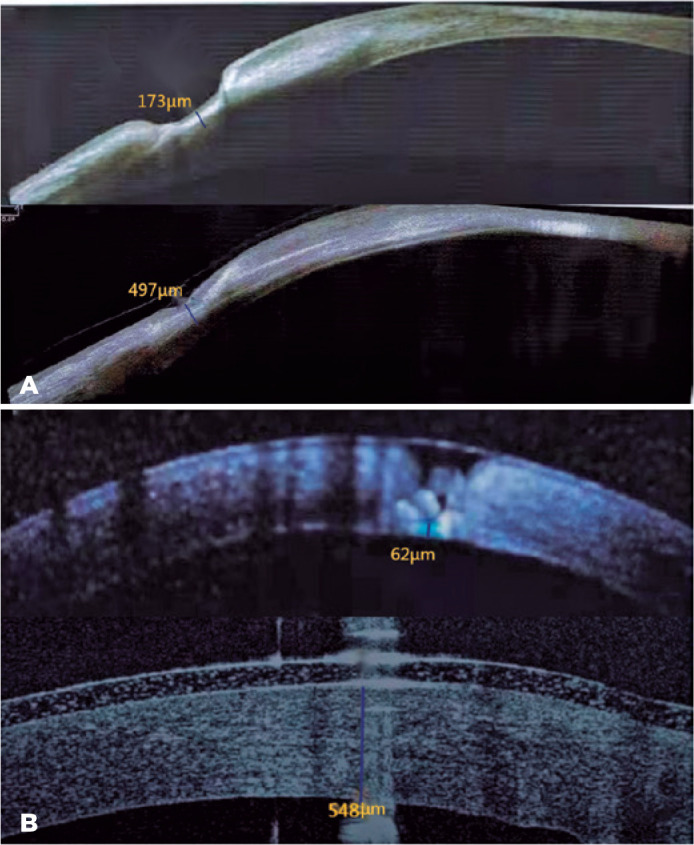
Corneal thickness was assessed by AS-OCT between the two groups. (A) Representative images of the AM-P group before operation (upper) and 12 months after operation (lower). (B) Representative images of the PKP group before operation (upper) and 12 months after operation (lower). AS-OCT, anterior segment optical coherence tomography; AM-P, wet bio-amniotic membrane plugging combined with transplantation; PKP, penetrating keratoplasty procedure.

The two-way ANOVA investigating corneal thickness found a significant group effect (F=7.811, p=0.007) and a significant time effect (F=1830, p<0.001). A significant interaction effect was also found between the time and group (F=20.89, p<0.001). The pairwise comparison showed that corneal thickness in the AM-P and PKP groups significantly increased 12 months after the operation (both p<0.001) (Figure 2). However, the corneal thickness of the PKP group was significantly higher than that of the AM-P group at postoperative month 12 (p<0.001) (Table 2).

### Analysis of controlling primary disease

**Figure 3:**
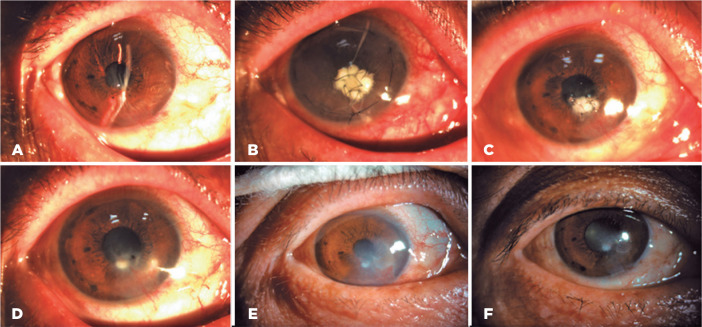
Representative images of successful treatment in the AM-P group. Before surgery (A) and 7 days (B), 1 month (C), 3 months (D), 6 months (E), and 12 months (F) after surgery. AM-P, wet bio-amniotic membrane plugging combined with transplantation.

**Figure 4:**
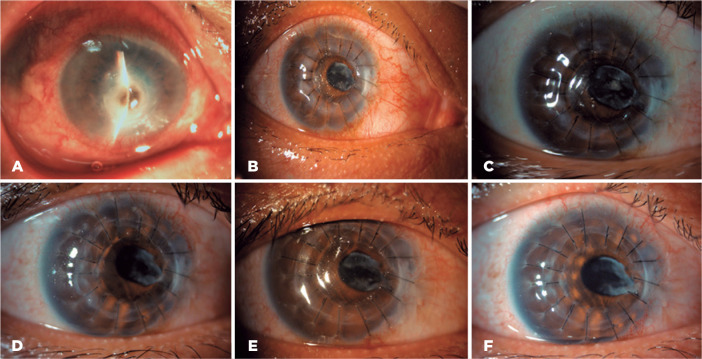
Representative images of successful treatment in the PKP group. Before surgery (A) and 7 days (B), 1 month (C), 3 months (D), 6 months (E), and 12 months (F) after surgery.

In the AM-P group, primary diseases were well controlled in 20 eyes (20/21, 95.2%), and 90% of the eyes recovered well after PKP (18/20). Furthermore, no difference in primary disease control was noted (p=0.481) (Table 3). Representative cases of graft success between the two groups are shown in figures 3 and 4.

### Postoperative complications

During the follow-up, complications between the two groups were not statistically different (p>0.999). Specifically, in the AM-P group, one eye had a secondary infection and one eye had high intraocular pressure. In the PKP group, graft immune rejection developed in only one eye (Table 3).

### Survival rate

**Figure 5:**
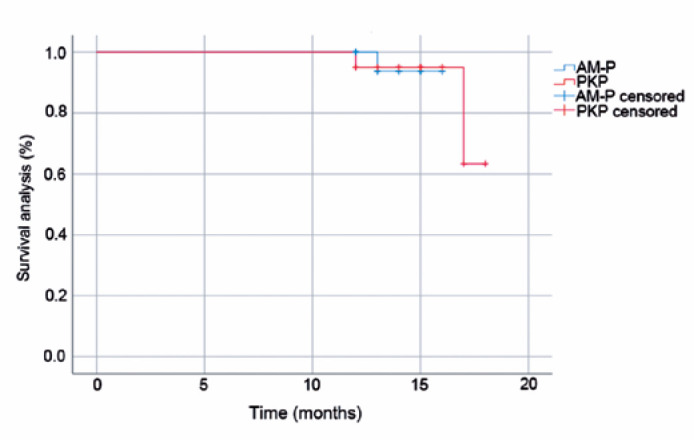
Survival rate in the AM-P and PKP groups. AM-P, wet bio-amniotic membrane plugging combined with transplantation; PKP, penetrating keratoplasty procedure.

With the last follow-up time as the node, primary disease recurrence and re-perforation of the corneal area were the endpoints in the AM-P group. In the PKP group, corneal graft rejection, failure, and primary di-sease recurrence were the endpoints. As shown in figure 5, no significant difference in the survival rate was found between the two groups (p=0.997).

## DISCUSSION

Corneal perforations are a potentially devastating late complications that can even cause precipitate corneal melting. For the treatment, PKP, amniotic membrane plugging, and amniotic membrane transplantation were reported effective for corneal perforations^(17-19)^. However, detailed reports on the treatment of small corneal perforations by the aforementioned methods are relatively limited, and their efficacy requires further investigation. In the present study, wet bio-amniotic membranes were adopted to evaluate their efficacy, and the results showed that AM-P can be used as an emergency treatment alternative to PKP for small corneal perforations.

PKP is a traditional treatment method for corneal perforations; however, it is limited by the availability of cornea donors^(4)^. With the wide application of amniotic membranes for treating ocular surface diseases, the indications are becoming more extensive. For instance, a study reported that the amniotic membrane can be deemed to be the main component supporting healing in the treatment of corneal perforations^(20)^. In addition, AM-P is effective in the treatment of severe ulceration and even corneal perforations, which have been confirmed^(21)^. In this study, a multilayer amniotic membrane was used to fill the defect to reconstruct the matrix and epithelium of corneal perforations, and then a larger amniotic membrane and corneal bandage mirror were used to cover the whole cornea and achieve rapid epithelialization. Correspondingly, AM-P has favorable efficacy for treating corneal perforations^(12,18)^. Moreover, the larger amniotic membrane covering the whole cornea provides mechanical protection for the corneal epithelium, allowing sufficient oxygenation and hydration of the epithelial cells and inhibiting inflammation^(22)^. Fortunately, the cornea was completely epithelialized in 95.2% of the patients without recurrence in the AM-P group, and no obvious difference was found when compared with PKP treatment. Thus, AM-P can be also used to treat small corneal perforations.

To our knowledge, vision recovery after surgery depends on corneal transparency. In this study, the BCVA was not markedly different between the two groups; however, the visual acuity in the PKP group improved more quickly than that in the AM-P group. The possible reason is that an integrated amnion exerts a progressive role in defect healing^(23)^. In addition, this study showed that the overall success rate in the AM-P group (95.2%) was similar or higher to that reported in previous clinical studies using amniotic membrane transplantation for the treatment of corneal ulcers or perforations^(18,24,25)^. The main purpose of the surgical treatment of corneal perforations is to re-epithelialize the defective corneal epithelium and stabilize the stromal thickness. Although the amniotic membrane does not provide absolute structural integrity of the cornea, it is sufficient to maintain the corneal shape and preserve the integrity of the eyeball. Furthermore, in the treatment of corneal perforations involving the optical axis, AM-P can provide sufficient time waiting for corneal donors and then performing PKP.

For patients with corneal perforations, special atten-tion should be paid to the formation of anterior chambers after surgery. If the aqueous humor is still leaking and the anterior chamber is not formed after the surgery, continuing the pressure bandage is necessary. In this study, anterior chambers were formed in the two groups within 3 days after the operation, whereas in the AM-P group, most of the anterior chambers formed on postoperative day 1. The time of anterior chamber formation may be related to postoperative intraocular pressure. However, since both groups wore contact lenses after surgery, the measurement of intraocular pressure after surgery was inaccurate, and no clear intraocular pressure value was recorded. Therefore, it is difficult to determine whether the intraocular pressure is related to the formation of the anterior chamber. Notably, a larger amniotic membrane covering the entire cornea provides mechanical protection for the corneal epithelium^(22)^. Therefore, AM-P can generate enough force to resist the pressure of the anterior chamber and prevent postoperative aqueous humor leakage and amniotic membrane plug outward expansion. In addition, the corneal thickness between the two groups increased significantly 12 months after the operation. Surprisingly, the postoperative corneal thickness of the PKP group was within the normal range. However, the postoperative corneal thickness of the AM-P group did not reach normal corneal thickness but enough to maintain normal corneal function, which was consistent with previous studies that have reported partial recovery of the corneal stromal thickness ^(16,26)^.

With the advances in corneal transplantation, postoperative complications are still perplexing patients and doctors. Overall, graft rejection after PKP treatment is a common cause of corneal graft failure^(27,28)^. Another retrospective survey reported that the 5-year and 10-year success rates in patients who underwent PKP for the first time were 90% and 82%, respectively, whereas the second transplantation has significantly lower survival rates of 53% and 41%, respectively ^(29)^. Moreover, AM-P is considered a good alternative to PKP, especially in acute cases with a high risk of graft rejection, as reported in a previous study^(16)^. In the AM-P group, a secondary infection occurred in one eye, which gradually improved after anterior chamber irrigation and matrix injection. Beyond that, intraocular pressure increased in one eye after the operation and decreased to normal after systemic and local hypotensive therapy. These findings suggested that AM-P may be a good choice for cases with a high risk of graft rejection.

This study has several limitations. First, the sample size was small, the follow-up time was short, and types of corneal perforations were limited. To further evaluate the efficacy of AM-P, a large randomized controlled study is needed. Second, as a retrospective study, there may be differences between the groups. Specifically, the etiology of perforation varied between the two groups, with more infections (viral and bacterial) in the PKP group and more foreign bodies in the AM-P group. However, given the small sample size, the difference was not statistically significant. Third, in this study, the anterior chamber formation time of the AM-P group was shorter than that of the PKP group. Nevertheless, determining whether the intraocular pressure is related to anterior chamber formation is difficult. In the future, we will further explore the relationship between intraocular pressure and anterior chamber formation.

AM-P is effective in the treatment of small corneal perforations and can be used as can be an emergency treatment alternative to PKP for small corneal perforations. For corneal perforations involving the optical axis, this technique can provide sufficient time to wait for the corneal donor and PKP.
